# A bivalent oral yeast vaccine displaying *Nocardia seriolae* FHA and LMBV MCP confers protection in largemouth bass (*Micropterus salmoides*)

**DOI:** 10.3389/fimmu.2026.1947573

**Published:** 2026-08-26

**Authors:** Jianfei Lu, Boge Gu, Pengzhen Yu, Jiong Chen

**Affiliations:** 1 Laboratory of Biochemistry and Molecular Biology, School of Marine Sciences, Ningbo University, Ningbo, China; 2 Key Laboratory of Aquacultural Biotechnology of Ministry of Education, Ningbo University, Ningbo, China

**Keywords:** bivalent vaccine, largemouth bass, largemouth bass ranavirus, *Nocardia seriolae*, yeast surface display

## Abstract

Largemouth bass ranavirus (LMBV) and *Nocardia seriolae* are major pathogens affecting largemouth bass (*Micropterus salmoides*), causing significant economic losses. In this study, recombinant yeasts expressing the fibronectin-binding protein A (FHA) of *N. seriolae* or the major capsid protein (MCP) of LMBV were constructed using the yeast surface display (YSD) system. Based on these recombinant strains, a bivalent oral yeast-based vaccine was developed and its protective efficacy was systematically evaluated. Oral administration of the vaccine induced specific antibodies against FHA and MCP in serum, as well as increased the transcription levels of adaptive immune genes (*IgM*, *IgT*, *MHC-II*) and innate immune genes (*IL-1β*, *TNF-α*, *IFN-1*, *Mx2*) in the hindgut, liver, and spleen. Importantly, the bivalent oral vaccine provided significant protection against both pathogens in largemouth bass, with relative percent survival (RPS) values of 56.7% against *N. seriolae* and 63.3% against LMBV. Meanwhile, the oral vaccine reduced pathogen loads and alleviated histopathological lesions. In summary, these findings suggest that the bivalent oral vaccine developed in this study could serve as a promising strategy for controlling *N. seriolae* and LMBV infections in largemouth bass aquaculture.

## 1 Introduction

Largemouth bass (*Micropterus salmoides*), a freshwater teleost native to North America, is an economically important aquaculture species, with an annual production of 880,000 tons in China (1). However, the outbreak of diseases continues to threaten the sustainable development of the largemouth bass aquaculture industry (2, 3). Largemouth bass ranavirus (LMBV) and *Nocardia seriolae* are two major pathogens affecting this species. LMBV is a DNA virus belonging to the genus *Ranavirus* and family Iridoviridae, typically causing ulceration in the skin and muscle, as well as necrotic hemorrhage in the liver, spleen, and kidney (4). *N. seriolae* is an aerobic Gram-positive bacterium belonging to the family *Nocardiaceae* and genus *Nocardia*, infection with this bacterium leads to skin ulceration and visceral nodules (5). To date, there is no commercial vaccine available against LMBV and *N. seriolae*.

Multivalent vaccines provide broader protection by targeting multiple pathogens in a single immunization, thereby reducing handling frequency, associated stress, and vaccination costs, without compromising immunogenicity or safety (6). Several studies have demonstrated the potential of multivalent vaccines in aquaculture (7–9). For example, a multivalent vaccine against *Streptococcus iniae*, *Lactococcus garvieae*, and *Yersinia ruckeri* in rainbow trout (*Oncorhynchus mykiss*) achieved relative percent survival (RPS) of 60%, 60%, and 70%, respectively, following injection immunization (7). These findings prompted us to develop a bivalent vaccine against the two major pathogens of largemouth bass. The major capsid protein (MCP) is the main component of the LMBV capsid, enveloping and protecting the viral genome, and has been widely used as a target for vaccine development against LMBV (10–13). Among the various proteins encoded by *N. seriolae*, fibronectin-binding protein A (FHA) has been shown to confer immunoprotection against *N. seriolae* infection in hybrid snakehead (*Channa maculata × Channa argus*) (14). Therefore, MCP and FHA were used to develop vaccines against LMBV and *N. seriolae*.

Fish live in water throughout their lives, making them particularly suitable for oral vaccination. The yeast surface display (YSD) system is a eukaryotic expression technology that displays heterologous proteins on the surface of *Saccharomyces cerevisiae*. The following advantages of this system make it suitable as a carrier for oral vaccines: first, yeast, as a food-grade microorganism, is non-pathogenic to animals and can protect antigens from degradation by gastric acid and enzymes in the digestive tract; second, the post-translational modification of antigens by yeast helps to improve their immunogenicity; in addition, the polysaccharides in the yeast cell wall can act as natural immune adjuvants to synergistically activate the mucosal and systemic immune responses of fish; finally, yeast fermentation has a low cost and is suitable for large-scale production, facilitating popularization and application (15–17). In fish, this platform has also been utilized for the development of vaccines against various pathogens, including LMBV (11), *Aeromonas hydrophila* (18), grass carp reovirus (GCRV) (19), cyprinid herpesvirus 2 (CyHV-2) (20, 21), and *Micropterus salmoides* rhabdovirus (MSRV) (22). These studies provide a theoretical basis for the construction of a bivalent yeast vaccine targeting LMBV and *N. seriolae* in the present study.

Here, we displayed FHA and MCP on the surface of *S. cerevisiae* separately and mixed the two recombinant yeasts at a ratio of 1:1 to formulate a bivalently displayed yeast vaccine. Its immunogenicity and protective efficacy after oral administration were evaluated by measuring serum antigen-specific antibodies, expression of immune-related genes, relative percent survival (RPS), pathogen loads, and histopathological changes.

## 2 Materials and methods

### 2.1 Fish and pathogens

Largemouth bass (6 ± 0.5 cm) were obtained from Suzhou, Jiangsu Province, China, and acclimated for 2 weeks at the aquaculture facility of Ningbo University, with the water temperature maintained at 22 ± 2°C throughout the acclimation period. After acclimation, fish were screened by PCR to confirm the absence of *N. seriolae* and LMBV. The fish were fed with commercial formula feed twice a day.

*N. seriolae* was provided by Prof. Xiaoli Huang (College of Animal Science and Technology, Sichuan Agricultural University, China). LMBV strain NB001 and *S. cerevisiae* strain EBY100 were maintained in our laboratory.

### 2.2 Prokaryotic expression and antibody preparation

The full-length ORFs of FHA (GenBank: AP017900.1) and MCP (GenBank: MN176304.1) were amplified using gene-specific primers (**Table 1**), and cloned into the pGEX-4T-3 vector by homologous recombination. The recombinant plasmids were subsequently transformed into *Escherichia coli* BL21 (DE3) and verified by PCR and sequencing. Protein expression was induced with 1 mM IPTG and harvested by centrifugation, followed by sonication. For the purification of FHA, the clarified supernatant was incubated with GST-tag Purification Resin (Beyotime, Hangzhou, China) for 4 h at 4°C with gentle shaking. After washing with PBS, the FHA was eluted using 10 mM reduced glutathione. For the purification of MCP, the pellet was washed twice with 2 M urea and solubilized in 4, 6, or 8 M urea. Protein expression and purity were evaluated by SDS-PAGE.

**Table 1:** Primers used in the present study.

Primer name	Accession number	Sequence (5'-3')	Usage
IgM	MN871984.1	CTCAATGACCCCCCCTAA CAAGCCAAGACACCAAAA	RT-qPCR
IgT	MZ388129.1	GAAGGTCAACAACGCTGAGTG TGTTGCTGGTCACATCTAGTCC	
IL-1β	XM038693206.1	CATGGAAGGCGGTAAAG TTGGGGCAACTGAGAGA	
MHC-II	XM038736591.1	TCACTACCTGCGAGTCAGGA CTTCAGCCTGGTGACAGGAG	
IFN-1	XM038709291.1	TCTTGCTGTGGTTCAGTCGG GCGATTAAGTGTGGGGTTG	
TNF-α	XM038707261.1	CTTCGTCTACAGCCAGGCAT TTTGGCACACCGACCTCACC	
Mx2	XM038696208.1	TAAAATGGCTGGGGTCGGGG CATTGCACGGAACGACCACC	
16S rRNA	JF810852.1	TCTCCACGACGCTTCACAC CGTAGGTGCGATGAAGATTGT	
MCP	MN176304.1	ACTTGCGTCACTCCTGTTGT AGGCTCAGGGCGTAAGAGTA	
18S rRNA	JQ896299.1	GGACACGGAAAGGATTGACAG GTTCGTTATCCGAATTAACCAGAC	
pYD1-MCP	MN176304.1	CGCGGATCCATGTCTTCTGTTACGGGT CCGGAATTCCAGGATGGGGAAACC	clone
pYD1-FHA	AP017900.1	CCCAAGCTTGGGGTGAGCGAGAACAAGGAC CGCGGATCCGCGAGCCGAATCGCTCGAAAC	
pGEX-MCP	MN176304.1	GGTTCCGCGTGGATCCATGTCTTCTGTTACGGGTTCTGG GATGCGGCCGCTCGAGCAGGATGGGGAAACCCATGGA	
pGEX-FHA	AP017900.1	GGTTCCGCGTGGATCCGTGAGCGAGAACAAGGACCCG GATGCGGCCGCTCGAGAGCCGAATCGCTCGA	

For antibody preparation, BALB/c mice were immunized intraperitoneally. Priming used 200 μg recombinant protein emulsified 1:1 with complete Freund’s adjuvant; boosters on days 14 and 21 employed 100 μg and 50 μg protein with incomplete Freund’s adjuvant, respectively. A final 10 μg antigen without adjuvant was given on day 28. Two days later, blood was collected, serum isolated, and IgG purified using Protein A + G magnetic beads. Purified antibodies were diluted 1:1 with glycerol and stored at -80°C.

### 2.3 Construction of recombinant *S. cerevisiae*

The FHA ORF products were digested with *Hind III* and *BamH I*, and the MCP ORF products were digested with *BamH I* and *EcoR I*. The purified products were ligated into the corresponding sites of the pYD1 vector using T4 DNA ligase (Takara, Shiga, Japan) and transformed into *S. cerevisiae*. The positive recombinant plasmids were screened by PCR and sequencing, and designated pYD1-FHA and pYD1-MCP, respectively. The primers were listed in **Table 1**. The recombinant plasmids were transformed into competent cells of *S. cerevisiae* EBY100 using the Super Yeast Transformation Kit (Coolaber, Beijing, China). The recombinant strains were designated as EBY100/pYD1-FHA and EBY100/pYD1-MCP. The recombinant strains were cultured in 2% glucose yeast nitrogen base-casamino acid (YNB-CAA) medium at 30°C and 180 rpm. When OD600 reached a range of 2-5, the cells were moved to YNB-CAA medium containing 2% galactose to induce expression of the FHA and MCP proteins.

### 2.4 Indirect immunofluorescence assay

The induced recombinant *S. cerevisiae* cells were harvested and washed three times with ice-cold PBS. Cells were incubated with an Anti-His monoclonal antibody (1:500; Sangon, Shanghai, China) overnight at 4°C with gentle rotation. After three washes with PBS, cells were incubated with an Alexa Fluor 488-labeled Goat Anti-Mouse IgG antibody (1:500; Beyotime) for 30 min. Cells were washed three times, mounted on glass slides, and examined using a fluorescence microscope (Nikon, Tokyo, Japan).

### 2.5 Western blot

Samples were separated by 12% SDS-PAGE and transferred onto 0.45 μm PVDF membranes (Millipore, Darmstadt, Germany). Membranes were blocked with 5% nonfat milk in PBST for 2 h at room temperature and incubated overnight at 4°C with mouse anti-FHA or anti-MCP polyclonal antibodies (1:1000 in PBST). After washing with PBST, membranes were incubated with an HRP-conjugated goat anti-mouse IgG (1:3000; Beyotime) for 2 h at room temperature. Immunoreactive bands were visualized using a supersensitive enhanced chemiluminescence (ECL) substrate according to the manufacturer’s instructions and imaged using an imaging system (Tanon, Shanghai, China).

### 2.6 Vaccination and challenge tests

2000 largemouth bass were randomly assigned to five groups (each group contains three parallel groups): PBS control, EBY100 (empty-vector) control, EBY100/pYD1-FHA (monovalent), EBY100/pYD1-MCP (monovalent), and EBY100/pYD1-FHA/MCP (bivalent). The bivalent vaccine was prepared by mixing equal volumes (1:1) of the two induced recombinant yeasts. Fish were orally gavaged with 100 μL of 1 × 10^9^ CFU/mL yeast or PBS as previously described (22). All fish were given gavage once a day for 7 d. The booster immunization, which was done in the same way as the initial immunization, was given after 7 d of cessation. Fish were anesthetized with 3-aminobenzoic acid ethyl ester methanesulfonate (MS-222; Sigma, St. Louis, USA) (0.1 g/kg). Nine fish per group were randomly sampled at 10, 20, and 30 d after the final immunization. Blood samples were collected via the caudal vein, clotted overnight at 4°C and centrifuged at 3000 × g for 15 min. The hindgut, spleen, and liver were also collected. All samples were stored at -80°C until use.

The challenge experiments were performed 30 d after the final immunization. For the *N. seriolae* challenge, 30 fish from each group except EBY100/pYD1-MCP were intraperitoneally injected with 50 μL of *N. seriolae* suspension (1 × 10^8^ CFU/mL), which was adopted from a previous study (23). For the LMBV challenge, 30 fish from each group except EBY100/pYD1-FHA were intraperitoneally injected with 50 μL of LMBV suspension (1 × 10^6.5^ TCID_50_/mL).

### 2.7 Detection of specific antibodies

Serum FHA- or MCP-specific antibody levels were measured by indirect ELISA. Briefly, 96-well plates were coated with serum (10 μg/mL; 100 μL/well) at 4°C overnight and then blocked with 5% BSA at 37°C for 2 h. Purified FHA or MCP protein (1 μg in 100 μL/well) was then added and incubated at 37°C for 1 h. Plates were incubated with mouse anti-FHA or anti-MCP polyclonal antibodies (1:500; 100 μL/well) at 37°C for 1 h, followed by HRP-conjugated goat anti-mouse IgG (1:5000) at 37°C for 1 h. Using TMB (Tiangen, Beijing, China) as a colorimetric substrate, absorbance at 450 nm was measured using a microplate reader (Potenov, Beijing, China).

### 2.8 Expression of immune-related genes

The expression of immune-related genes including *immunoglobulin M* (*IgM*), *immunoglobulin T* (*IgT*), *major histocompatibility complex class II* (*MHC-II*), *interleukin-1β* (*IL-1β*), *tumor necrosis factor α* (*TNF-α*), *myxovirus resistance 2* (*Mx2*), *interferon 1 (IFN-1)*, and *18S rRNA* was detected by RT-qPCR as previously reported (24). Total RNA was extracted using TRIzol (Takara). cDNA was synthesized using HiScript^®^ III All-in-one RT SuperMix Perfect for qPCR (Vazyme, Nanjing, China). RT-qPCR was performed using 2 × Taq Pro Universal SYBR qPCR Master Mix (Vazyme) on ABI QuantStudio 3 Real-Time PCR System. *18S rRNA* was used as the endogenous control, the premier was listed in **Table 1**, and relative gene expression was calculated using the 2^-ΔΔCt^ method.

### 2.9 Pathogen load

Hindgut, liver, and spleen tissues were collected after challenge, total RNA was extracted using TRIzol reagent, and then reverse-transcribed into cDNA. The pathogen loads of *N. seriolae* and LMBV were quantified by qPCR as in section 2.8. The primers were listed in **Table 1**.

### 2.10 Histopathology

Tissues were rinsed with PBS and fixed in 4% paraformaldehyde at room temperature for 48 h. After fixation, the samples were embedded in paraffin and sectioned. The paraffin-embedded tissues were sectioned transversely at a thickness of 5 μm and stained with hematoxylin and eosin (H&E). The stained sections were examined and photographed under a light microscope.

### 2.11 Data analysis

Statistical analyses and figure generation were performed using GraphPad Prism (version 10.1). Data are presented as mean ± standard deviation (SD). Comparisons among groups were analyzed by one-way analysis of variance (ANOVA). *P* < 0.05 was considered statistically significant, and *P* < 0.01 was considered highly significant.

## 3 Results

### 3.1 Prokaryotic and eukaryotic expression of FHA and MCP

**Figure 1:**
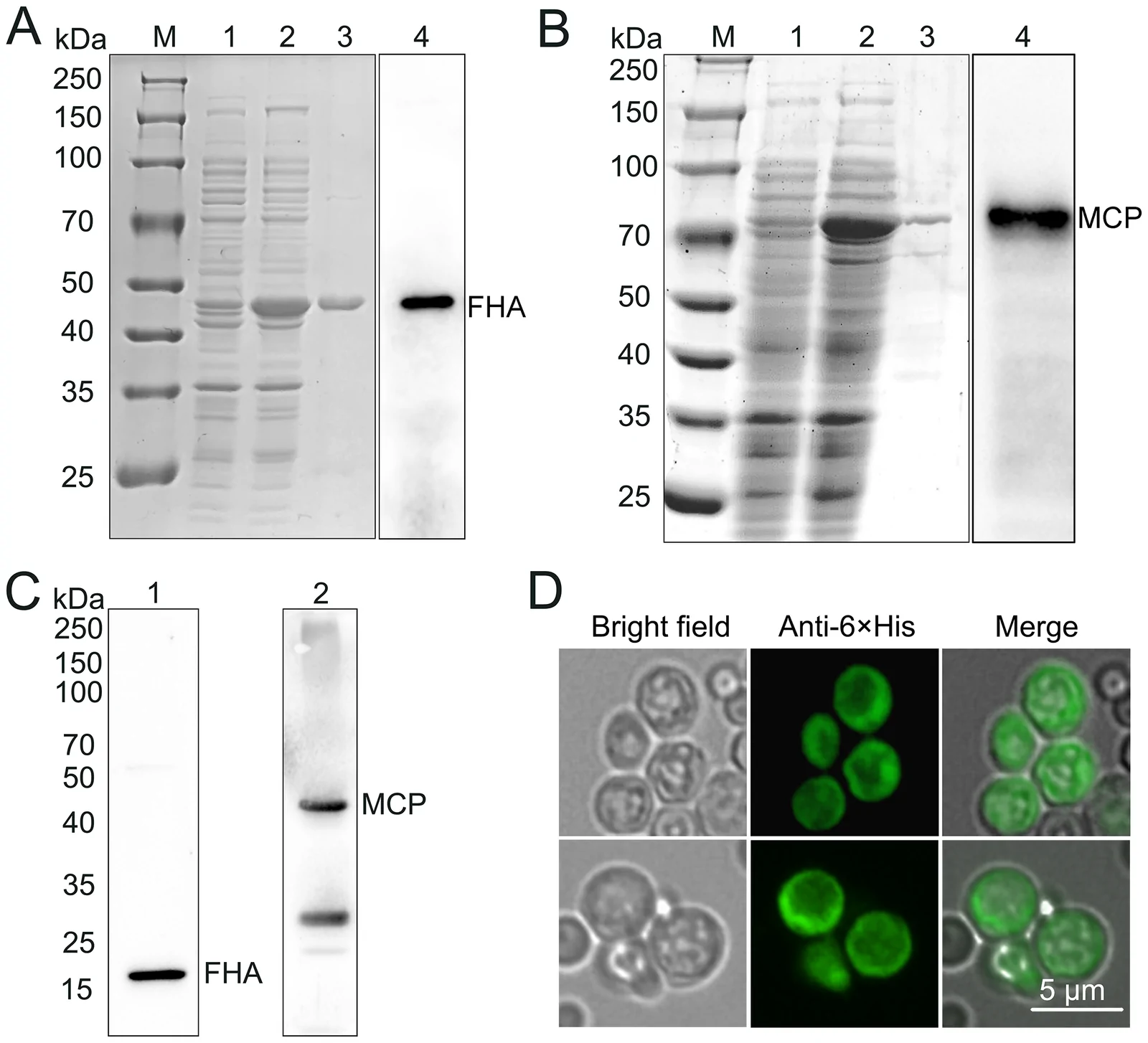
Prokaryotic and eukaryotic expression of FHA and MCP. **(A, B)** Prokaryotic expression and antibody preparation of FHA **(A)** and MCP **(B)**. The left shows SDS-PAGE analysis of corresponding protein, and the right shows WB analysis of FHA or MCP antibodies. M, protein marker; lane 1, uninduced sample; lane 2 and 4, IPTG induced sample; lane 3, purified FHA or MCP protein. **(C)** WB analysis of FHA and MCP in recombinant *S. cerevisiae*. **(D)** IFA analysis of EBY100/pYD1-FHA (top) and EBY100/pYD1-MCP (bottom). Bar = 5 μm.

For the prokaryotic expression and antibody preparation of FHA and MCP. The expression of FHA and MCP was confirmed by SDS-PAGE. As shown in **Figures 1A, B**, distinct bands corresponding to approximately 43.9 kDa (FHA) and 77.3 kDa (MCP) were detected in induced samples, but were absent in uninduced controls. Purified FHA and MCP each displayed a single prominent band at the predicted molecular weight, confirming successful expression and purification (**Figures 1A, B**). Meanwhile, the prepared polyclonal antibodies can also detect a single band of the corresponding protein, indicating successful preparation of the polyclonal antibodies.

**Figure 2:**
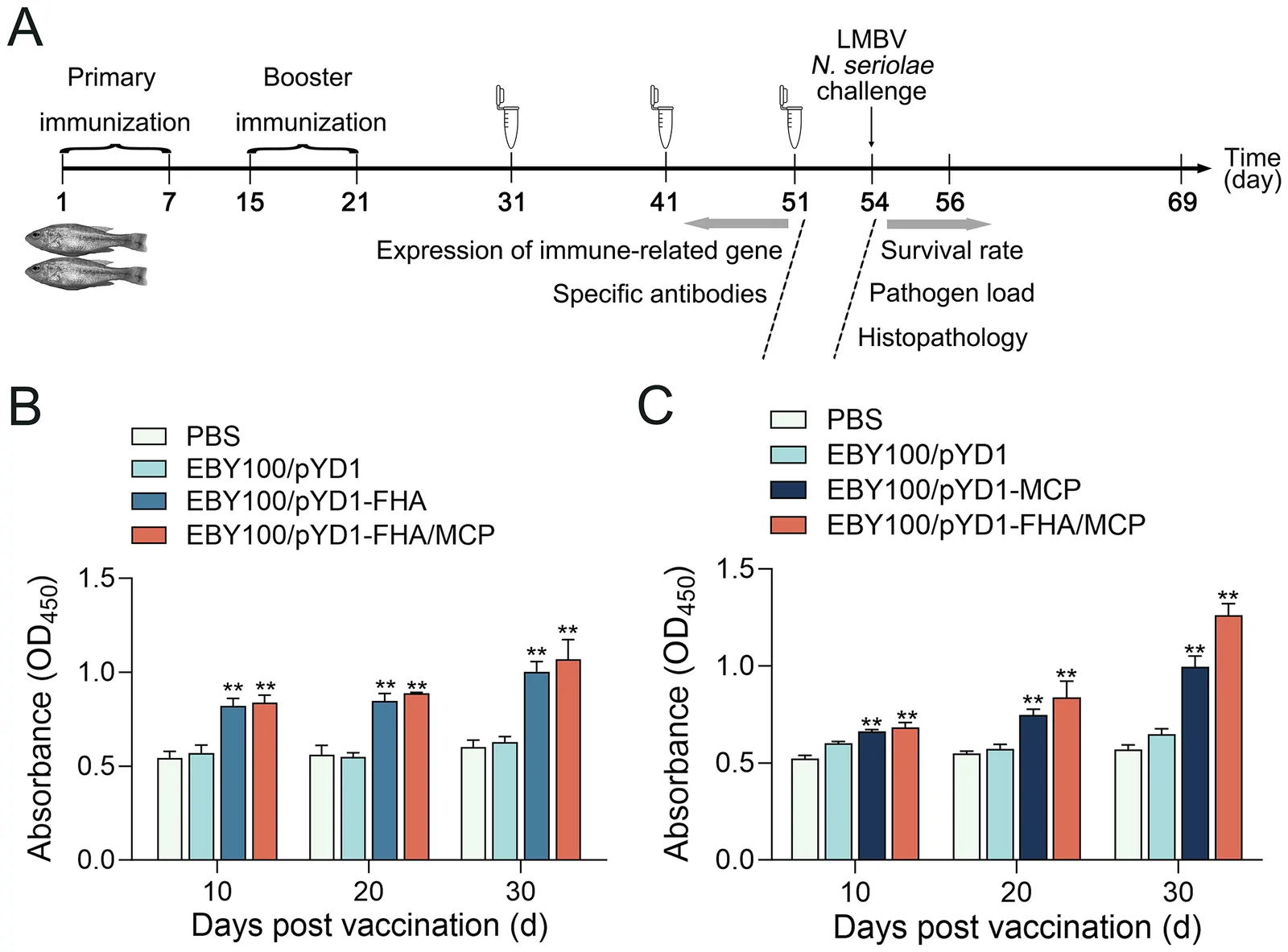
Experimental design and detection of specific antibodies. **(A)** Schematic diagram of the immunization in this study. **(B)** The detection of FHA-specific antibodies by ELISA. **(C)** The detection of MCP-specific antibodies by ELISA. Data were presented as mean ± SD (n = 3). *P < 0.05, **P < 0.01.

For the expression of FHA or MCP in recombinant *S. cerevisiae*. In the WB analysis, bands of approximately 16.8 kDa (FHA) and 50.1 kDa (MCP) were detected, consistent with the expected molecular weights (**Figure 1C**). In the IFA, green fluorescence was observed (**Figure 1D**). These results confirm the successful expression of FHA and MCP in *S. cerevisiae*. The prepared oral vaccines were immunized according to the procedure shown in **Figure 2A**.

### 3.2 FHA and MCP-specific antibodies

FHA and MCP-specific antibodies in serum were measured by ELISA at 10, 20, and 30 d post vaccination (dpv). FHA antibody levels increased over time in both the EBY100/pYD1-FHA and EBY100/pYD1-FHA/MCP groups, with peak levels at 30 dpv (**Figure 2B**). MCP antibody was not detected at 10 dpv and was increased over time in both the EBY100/pYD1-MCP and EBY100/pYD1-FHA/MCP groups, with peak levels at 30 dpv (**Figure 2C**).

### 3.3 Expression of immune-related genes

**Figure 3:**
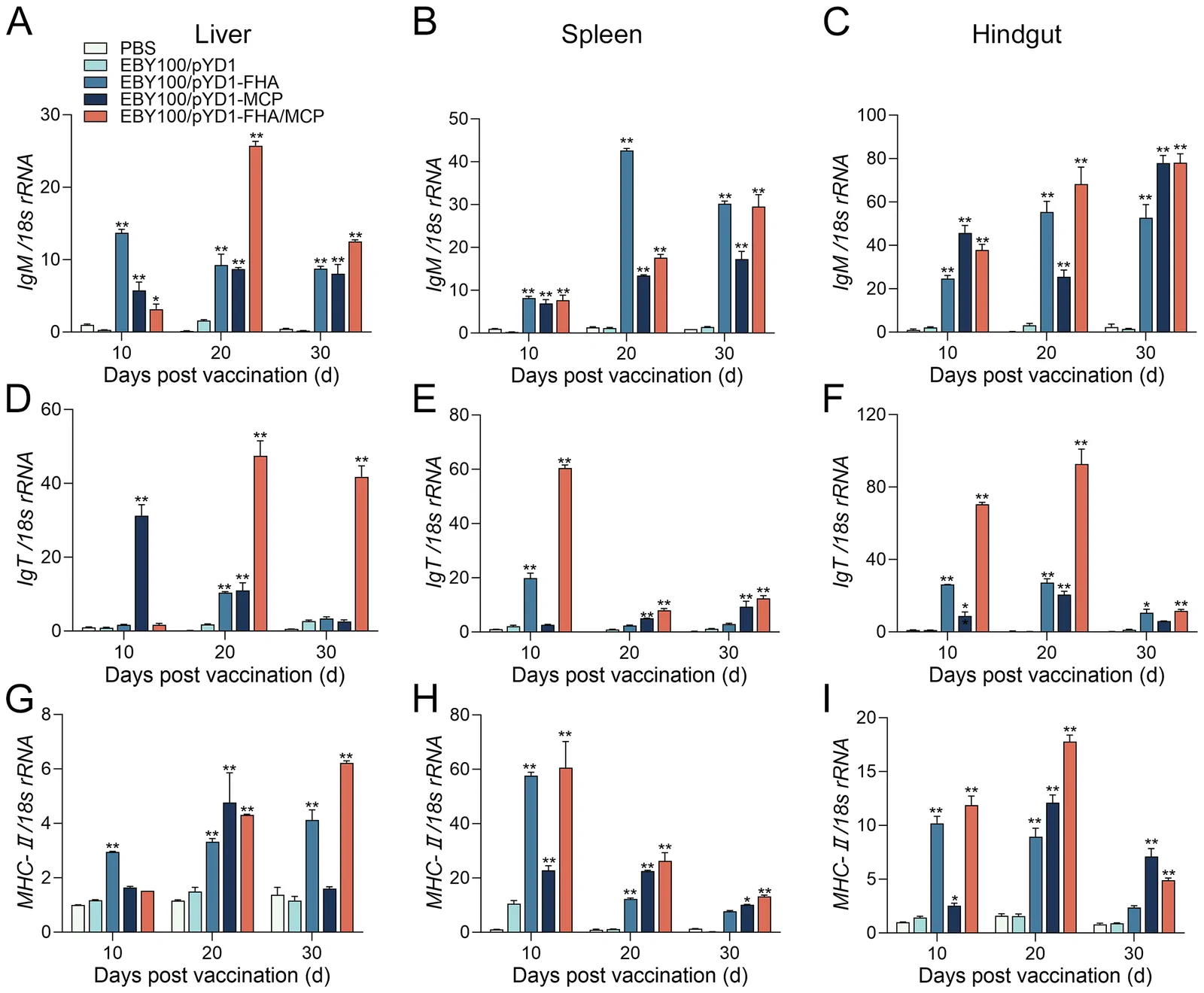
Relative expression of *IgM*, *IgT*, and *MHC-II* in the liver **(A, D, G)**, spleen **(B, E, H)**, and hindgut **(C, F, I)** after vaccination. Data were presented as mean ± SD (n = 3). *P < 0.05, **P < 0.01. All significance analyses were conducted using the PBS control group as a reference.

To assess vaccine-elicited immune responses, we quantified the transcripts of *IgM*, *IgT*, and *MHC-II* in the liver, spleen, and hindgut at 10, 20, and 30 dpv. Across one or more time points, the expression of *IgM*, *IgT*, and *MHC-II* was increased relative to the PBS group. The strongest induction of *IgM* and *IgT* was observed in the hindgut, reaching 53.00- and 27.08-fold in EBY100/pYD1-FHA, 74.65- and 20.46-fold in EBY100/pYD1-MCP, and 74.74- and 92.20-fold in the bivalent group, respectively (**Figure 3**). Meanwhile, *MHC-II* upregulation was most pronounced in the spleen (~57.58-fold in EBY100/pYD1-FHA, 22.71-fold in EBY100/pYD1-MCP, and 60.49-fold in the bivalent group), exceeding that in the hindgut.

**Figure 4:**
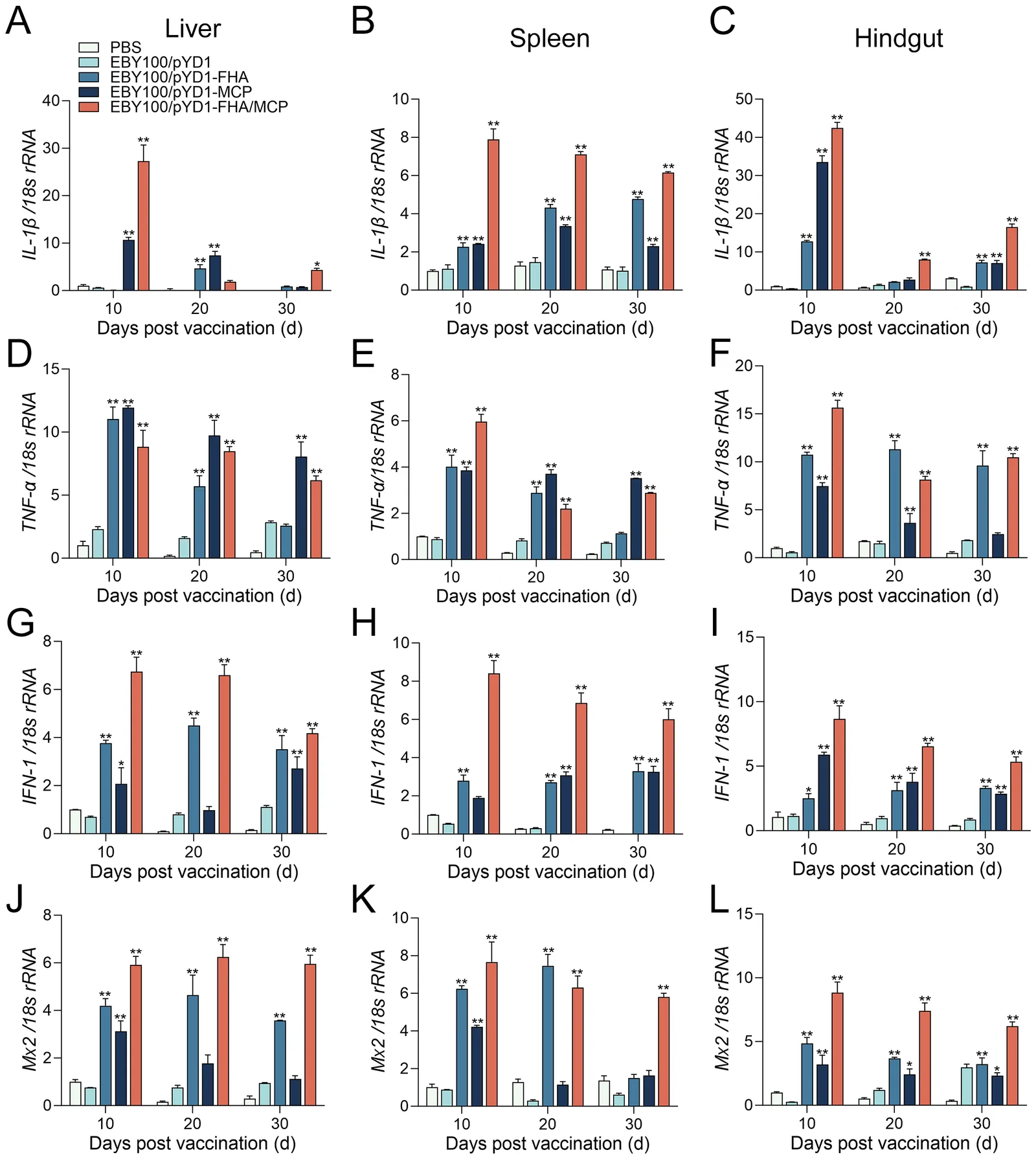
Relative expression of *IL-1β*, *TNF-α*, *IFN-1*, and *Mx2* in the liver **(A, D, G, J)**, spleen **(B, E, H, K)**, and hindgut **(C, F, I, L)** after vaccination. The expression levels of all genes were calculated relative to the PBS control group. Data were presented as mean ± SD (n = 3). *P < 0.05, **P < 0.01. All significance analyses were conducted using the PBS control group as a reference.

We next examined the expression of *IL-1β*, *TNF-α*, *IFN-1*, and *Mx2*. These transcripts were generally elevated in monovalent and bivalent groups compared with the PBS group across the assessed time points (**Figure 4**). Collectively, these data indicate that both monovalent and bivalent enhance adaptive and innate immune gene expression.

### 3.4 Protective efficacy of the bivalent vaccine

**Figure 5:**
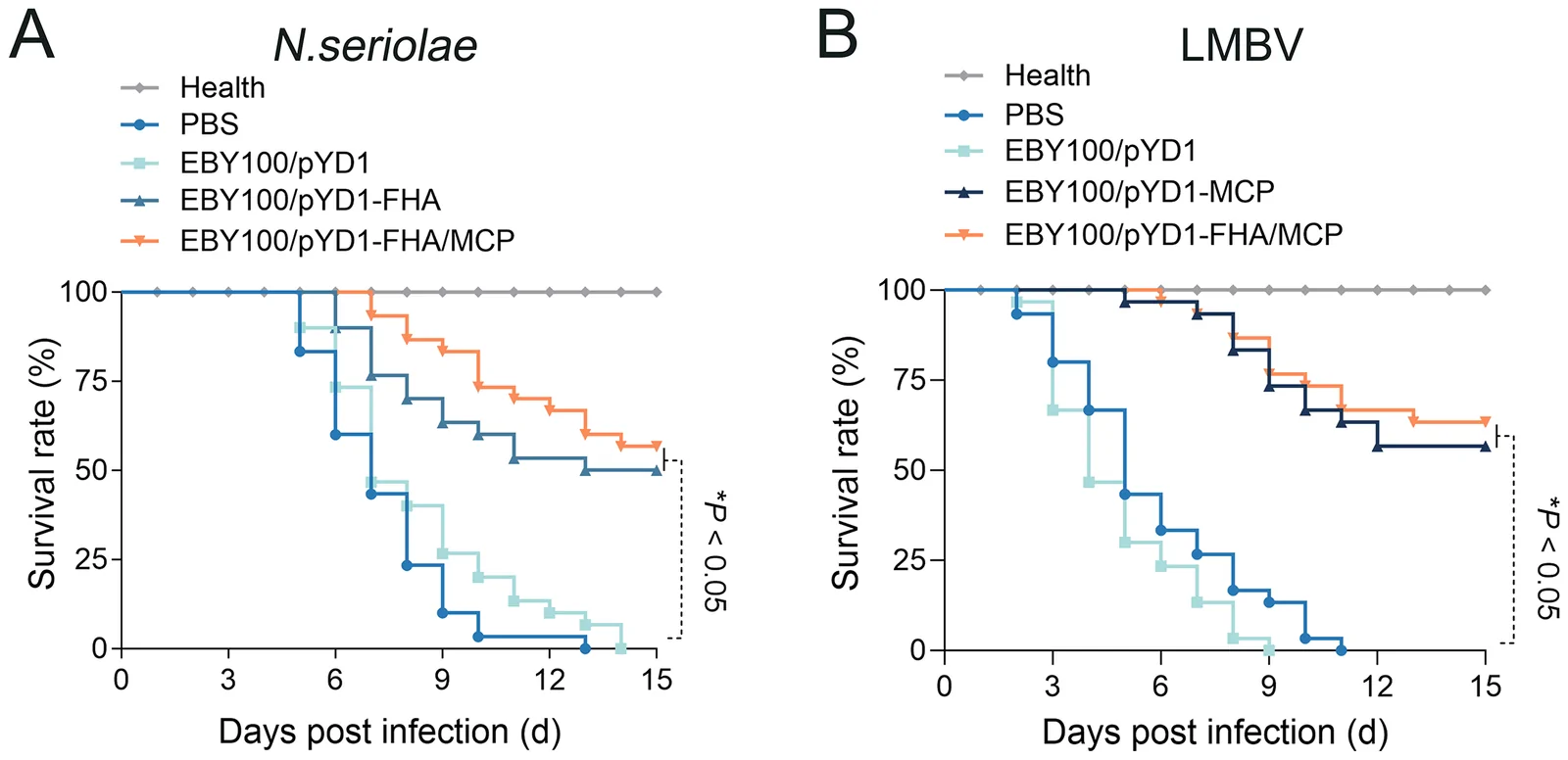
Kaplan-Meier analysis of cumulative mortality in largemouth bass following challenge with *N. seriolae* **(A)** or LMBV **(B)**. Survival curves were analyzed using the log-rank test with pairwise comparisons. n = 3; *P < 0.05.

Protective efficacy was evaluated by challenging vaccinated fish with *N. seriolae* or LMBV at 33 dpv. Following *N. seriolae* challenge, the death of fish in EBY100/pYD1 and PBS began at 5 d post infection (dpi) and reached 100% by 13 dpi and 14 dpi. The death onset occurred at 6 dpi in the FHA monovalent group and 7 dpi in the bivalent group, corresponding to RPS of 50.0% and 56.7%, respectively (**Figure 5A**). No significant difference in survival rate between the bivalent and FHA monovalent groups (*P* = 0.3861). Following LMBV challenge, the dead fish in the EBY100/pYD1 and PBS groups began at 2 dpi and reached 100% by 11 and 9 dpi, respectively. By contrast, the death onset was delayed to 5 dpi in the MCP monovalent group and to 6 dpi in the bivalent group, resulting in RPS of 56.7% and 63.3%, respectively (**Figure 5B**). Similarly, survival rate did not differ significantly between the bivalent and MCP monovalent groups (*P* = 0.6028).

### 3.5 The bivalent vaccine reduces LMBV and *N. seriolae* loads

**Figure 6:**
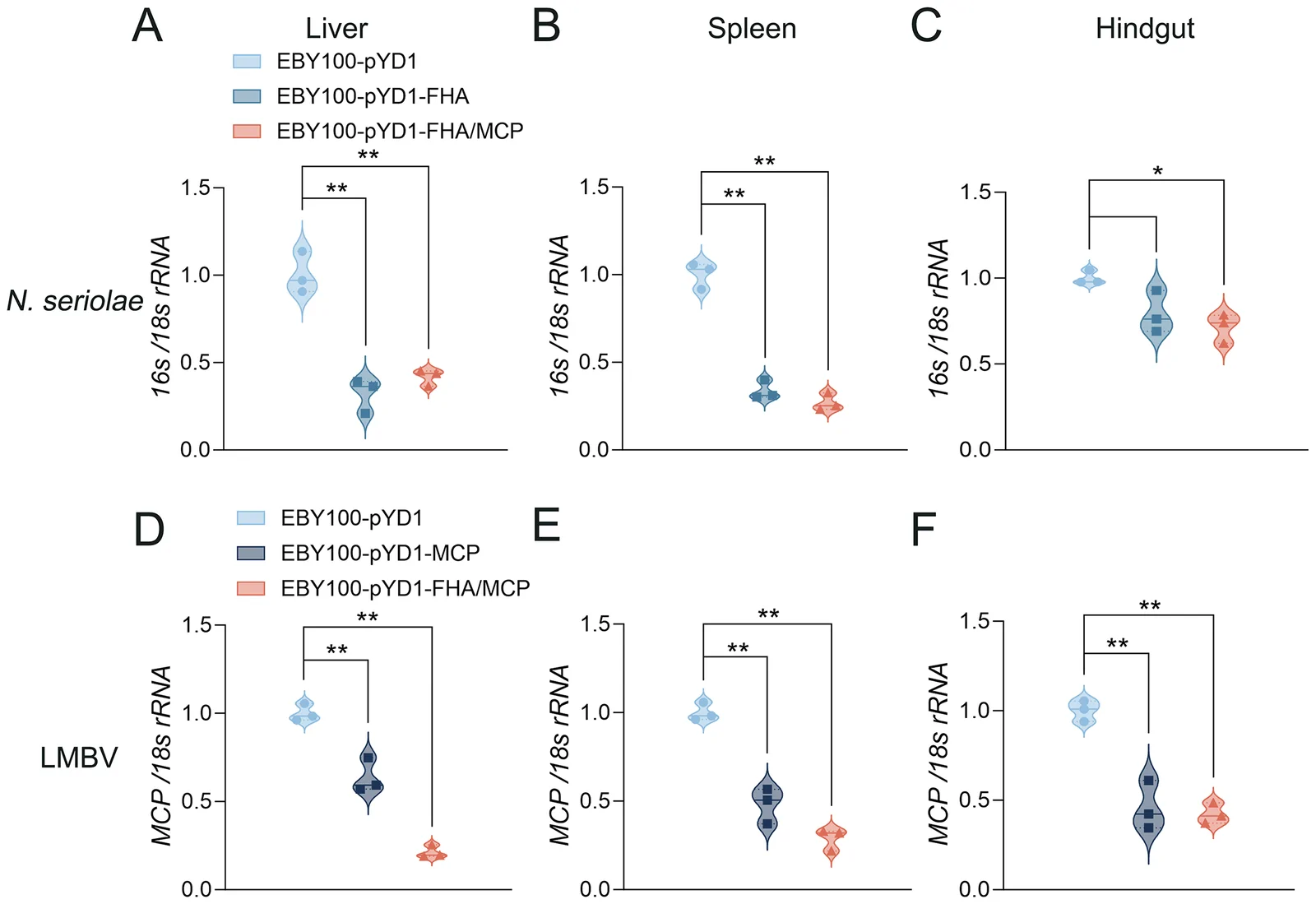
Pathogen loads in largemouth bass tissues following *N. seriolae* or LMBV challenge. **(A–C)** *N. seriolae* loads. At 4 dpi with *N. seriolae*, the liver, spleen, and hindgut tissues were sampled. **(D–F)** LMBV loads. At 2 dpi with LMBV, the liver, spleen, and hindgut tissues were sampled. The bacterial and viral loads were quantified by RT-qPCR. n = 3; *P < 0.05, **P < 0.01.

To investigate the effect of vaccination on pathogen load, tissue samples were collected at 4 dpi (*N. seriolae*) or 2 dpi (LMBV). Following *N. seriolae* challenge, bacterial loads in the EBY100/pYD1-FHA and EBY100/pYD1-FHA/MCP groups were decreased compared with the EBY100/pYD1 group (**Figures 6A–C**). After the LMBV challenge, viral loads in both the EBY100/pYD1-MCP and EBY100/pYD1-FHA/MCP groups were also reduced relative to the EBY100/pYD1 group (**Figures 6D–F**). Overall, vaccination reduced *N. seriolae* and LMBV loads in liver, spleen, and hindgut tissues.

### 3.6 Vaccination alleviates histopathological lesions induced by *N. seriolae* and LMBV

**Figure 7:**
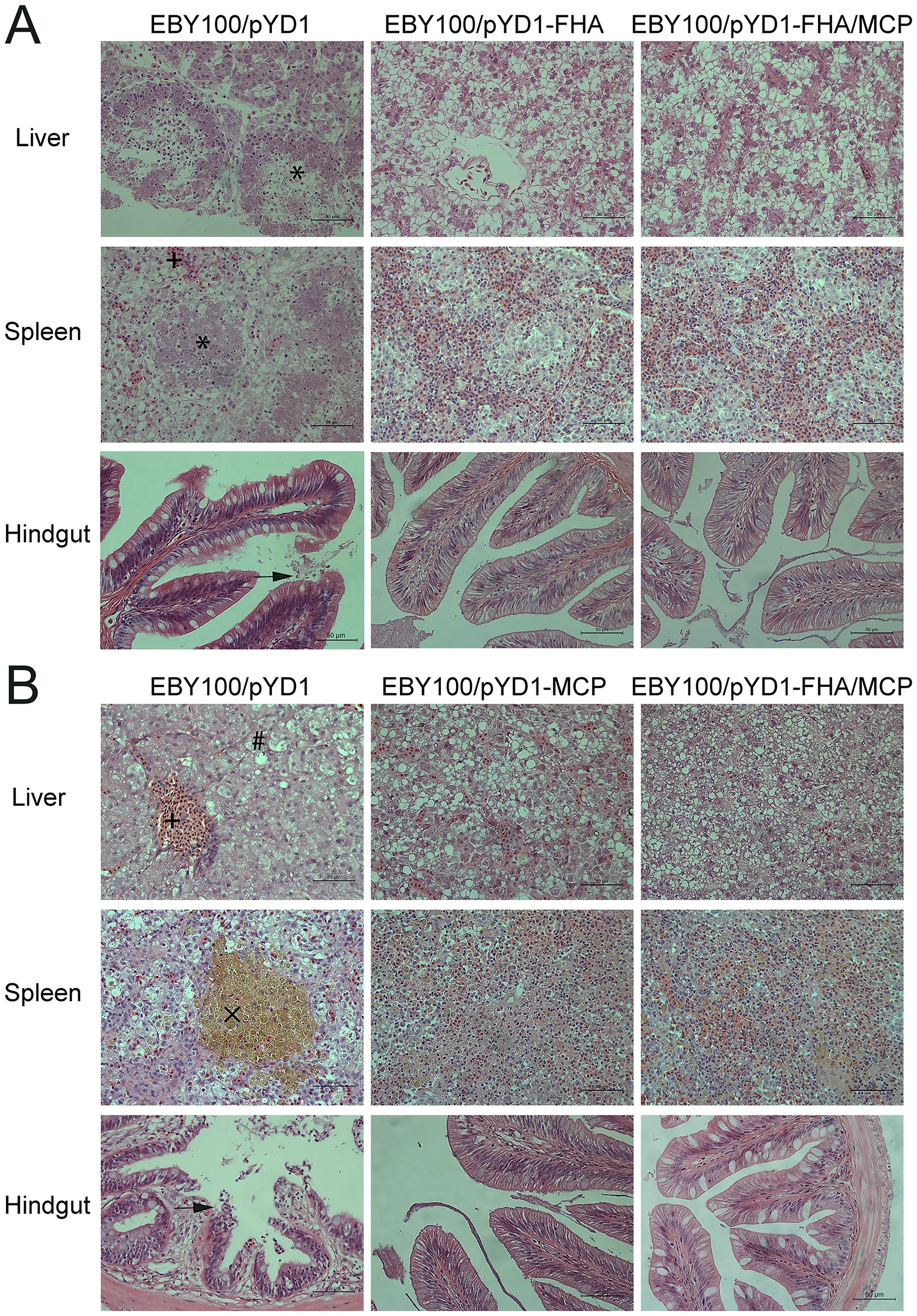
Vaccination alleviates histopathological lesions induced by *N. seriolae* **(A)** or LMBV **(B)**. (*) indicates granulomatous lesions, (+) indicates venous congestion, (→) indicates epithelial shedding and structural disruption, (#) indicates necrotic foci, (×) indicates hemosiderin deposition. Scale bar = 50 μm.

To evaluate the effect of vaccination on *N. seriolae*-induced tissue damage, histopathological changes in tissues were examined at 4 dpi. The results showed that the EBY100/pYD1 group exhibited epithelial shedding and structural disruption in the hindgut, as well as necrotic foci and granulomatous lesions in the liver and spleen. In contrast, the EBY100/pYD1-FHA and EBY100/pYD1-FHA/MCP groups retained relatively intact tissue structures, with no or only mild lesions observed in the hindgut, liver, and spleen (**Figure 7A**).

At 2 d post LMBV challenge, the EBY100/pYD1 group similarly showed epithelial shedding and structural disruption in the hindgut, accompanied by obvious central venous congestion and cellular necrosis in the liver, as well as hemosiderin deposition in the spleen. In contrast, only mild or no obvious pathological changes were observed in the EBY100/pYD1-MCP and EBY100/pYD1-FHA/MCP groups (**Figure 7B**). Overall, compared with control, both monovalent and bivalent vaccination reduced the severity of tissue damage associated with LMBV and *N. seriolae* infection.

## 4 Discussion

The aquaculture industry of largemouth bass is increasingly threatened by various pathogens, among which LMBV and *N. seriolae* cause large-scale mortality and significant economic losses (2, 25). Given the absence of commercial vaccines against these two pathogens, the development of efficient and practical dual-targeted prevention strategies is urgently needed. Based on the YSD technology platform, this study successfully constructed a bivalent oral yeast vaccine, providing an innovative approach for the simultaneous control of these two major pathogens.

Oral vaccines are a research hotspot for fish vaccines, with the main advantages of significantly reducing labor intensity, minimizing fish stress, and lowering costs. Expressing antigens on the surface of *S. cerevisiae* not only enables effective antigen delivery but also leverages the probiotic properties of yeast to enhance immunogenicity, which has become one of the mainstream strategies for oral vaccine development (15). MCP, a key protective antigen of LMBV, has been utilized in DNA vaccines, achieving an RPS of 89.5% via injection (26, 27). Similarly, protein vaccines based on MCP fragments have achieved an RPS of up to 88.89% following injection (28). FHA, an important virulence factor of *N. seriolae*, has been shown to confer immune protection in hybrid snakehead, with an RPS of 62.64% via injection (14). In this study, both monovalent and bivalent vaccines showed protective effects against the corresponding pathogen infections after oral administration, highlighting the utility of YSD technology in developing oral vaccines for fish. However, this study observed that the RPS of oral vaccines was generally lower than that of injection immunization in other studies (14, 28, 29). This phenomenon is mainly attributed to two aspects: first, the extreme environment of the fish gastrointestinal tract (such as gastric acid and enzymes) may damage part of the yeast vector or antigen structure, leading to a decrease in antigen bioavailability; second, the antigen copy number displayed by the YSD system and the antigen presentation efficiency have inherent limitations in the oral route (15, 30). Future efforts should focus on optimizing yeast carriers, developing acid-resistant formulations, or designing novel delivery systems to overcome these bottlenecks and further enhance the efficacy of oral vaccines.

Although bivalent vaccines offer advantages such as targeting two pathogens simultaneously, improving immunization efficiency, and reducing vaccination costs, they may also have disadvantages, including antigenic competition, imbalanced immune responses, or complex manufacturing processes (31, 32). In this study, the RPS of the developed bivalent vaccine was slightly higher than that of the monovalent vaccines. Specifically, the RPS against *N. seriolae* infection was 56.7% and 50.0% for the bivalent and monovalent vaccines, respectively, while the RPS against LMBV infection was 63.3% and 56.7%, respectively. Notably, at the same total vaccine dose, the amount of each component in the bivalent vaccine was half that of the monovalent counterpart. Despite this dose reduction, the bivalent vaccine achieved comparable protective efficacy, suggesting that the bivalent formulation enhances the immunogenicity of each component. This phenomenon is commonly observed in multivalent vaccines. For instance, in an inactivated bivalent vaccine of *A. hydrophila* and *S. iniae*, the RPS of the bivalent vaccine was higher than that of the monovalent vaccines (33). We speculate that this synergistic effect may result from the mutual promotion of the two antigens during the immune stimulation process, which jointly activate a broader population of immune cells or a stronger inflammatory microenvironment. This finding provides an important insight for the development of efficacious multivalent vaccines in aquaculture, although the underlying molecular mechanisms warrant further investigation.

The protective efficacy of a vaccine ultimately depends on the intensity and quality of the immune response it induces. In this study, we analyzed the protective mechanisms of the bivalent oral vaccine from the perspectives of both innate and adaptive immunity. IgM serves as the core molecule of humoral immunity in teleosts, while IgT plays a key role in mucosal immunity, particularly within the intestine (34). MHC-II molecules are responsible for antigen presentation and initiating specific T-cell immune responses (35). This study found that the bivalent vaccine significantly upregulated the expression levels of *IgM*, *IgT*, and *MHC-II* in the liver, spleen, and hindgut. This suggests that the bivalent vaccine effectively activates both systemic and mucosal humoral immunity, providing a critical immune barrier for pathogen clearance. In addition, innate immunity constitutes the first line of defense against pathogen invasion. TNF-α and IL-1β are important pro-inflammatory cytokines, and IFN-1 and Mx2 also play key roles in the disease resistance process (36–38). In this study, the expression of *TNF-α*, *IL-1β*, *IFN-1*, and *Mx2* was induced by vaccination, further confirming that the bivalent vaccine can stimulate innate immune responses. Collectively, these findings indicate that the bivalent vaccine confers protection through specific immunity and nonspecific immunity, thereby providing faster and broader protection to the fish.

## 5 Conclusion

In conclusion, this study developed a bivalent yeast vaccine by displaying FHA and MCP on the surface of *S. cerevisiae* and mixing the two recombinant strains. Oral immunization improved the survival rate of largemouth bass infected with *N. seriolae* and LMBV, and induced robust adaptive and innate immune responses. In addition, oral immunization effectively inhibited the loads of *N. seriolae* and LMBV and the tissue damage caused by these pathogens. These findings suggest that the bivalent oral vaccine based on YSD technology is a promising strategy for the simultaneous control of *N. seriolae* and LMBV infections in largemouth bass aquaculture.

## Data Availability

The original contributions presented in the study are included in the article/supplementary material. Further inquiries can be directed to the corresponding author.
